# Exploration of vanoxerine analogues as antibacterial agents

**DOI:** 10.1038/s41429-024-00781-9

**Published:** 2024-10-15

**Authors:** Alexander D. H. Kingdon, Holly V. Adcock, Eleni-Marina Kasimati, Philip Craven, Willem van Schaik, Liam R. Cox, Gurdyal S. Besra

**Affiliations:** 1 School of Biosciences, University of Birmingham, Birmingham, B15 2TT UK; 2 School of Chemistry, University of Birmingham, Birmingham, B15 2TT UK; 3 Present Address: Department of Tropical Disease Biology, Liverpool School of Tropical Medicine, Liverpool, L3 5QA UK

**Keywords:** Microbiology, Molecular biology

## Abstract

*Mycobacterium tuberculosis* is a bacterial pathogen, responsible for approximately 1.3 million deaths in 2022 through tuberculosis infections. The complex treatment regimen required to treat tuberculosis and growing rates of drug resistance, necessitates the development of new anti-mycobacterial agents. One approach is to repurpose drugs from other clinical applications. Vanoxerine (GBR 12909) was previously shown to have anti-mycobacterial activity, through dissipating the membrane electric potential and hence, cellular energetics. Several vanoxerine analogues were synthesised in this study, which exhibited a range of activities against mycobacteria and enterococcus. All active analogues had similar impacts on the membrane electric potential and inhibition of ethidium bromide efflux. The most active compound displayed reduced inhibitory activity against the known human target of vanoxerine, the dopamine transporter. This work has identified a promising analogue, which could provide a starting point for further medicinal chemistry and drug development efforts to target mycobacteria.

## Introduction

*Mycobacterium tuberculosis* is a deadly pathogen, that has plagued humanity for thousands of years, and led to the deaths of approximately 1.3 million individuals in 2022 [1]. In the same year, tuberculosis (TB) infections occurred in an estimated 10.6 million individuals, with low- and middle-income countries bearing the largest burden of disease [1]. The COVID-19 pandemic hindered both diagnostic and treatment programmes in many countries, resulting in more infections in 2021 and 2022 than in previous years [1]. Additional resources and investments are required to combat this global pandemic, including new drug discovery initiatives.

The current treatment regimen for drug-susceptible TB infections comprises four drugs, isoniazid, rifampicin, ethambutol, and pyrazinamide [1], and has remained unchanged for 60+ years [2]. This treatment regimen places large financial and health burdens on patients. The treatment success rate following this 6-month regimen was 88% in 2021, while multi-drug resistant TB, only had a treatment success rate of 63% in 2020 [1]. It remains to be seen if the newly introduced bedaquiline, pretomanid, linezolid and moxifloxacin 6-month treatment regimen will improve these patient outcomes [3]. Drug resistance is a growing issue, with clinical resistance identified against all currently used drugs [4–6]. To supplement the current drug discovery pipeline, it has been suggested that at least twenty novel anti-mycobacterial drugs need to enter clinical evaluation as soon as possible [7]. Additional discovery projects are required to meet these demands and produce new treatments for TB infections, with a focus on novel mechanisms of action to avoid cross-resistance.

Repurposing drugs for the treatment of TB is a common approach, with the fluoroquinolones and linezolid demonstrating the success of this strategy. Approximately 25% of the drugs undergoing clinical trials for TB are repurposed therapeutics [8]. Vanoxerine (GBR 12909) is a synthetic compound which has been developed for the treatment of cocaine dependence and arrhythmia [9, 10]. It has not entered clinical use, as its development was stopped after phase I, and during phase III, clinical trials for these two indications [11, 12]. Despite favourable tolerance by healthy volunteers [10, 13, 14], vanoxerine caused ventricular pro-arrhythmia in three patients with structural heart disease during a phase III clinical trial, resulting in the termination of the trial [11]. Further testing is required to evaluate if it is safe to use for other clinical applications [15]. Vanoxerine analogues were synthesised during its development as a dopamine transport inhibitor; whilst less effective for that application, these agents could represent alternative antibacterial compounds [16–19]. In particular, the decanoyl ester of vanoxerine was shown to eliminate cocaine-maintained behaviour in rhesus monkeys for one month, following a single injection, suggesting an extended half-life [16].

The anti-mycobacterial activity of vanoxerine has been reported, with MIC_99_ values against *M. tuberculosis* H37Rv of 6.5 and 14 µg ml^−1^ [20, 21]. AroB, an essential protein involved in aromatic amino acid synthesis, was suggested to be the mycobacterial target of vanoxerine [21]; however, this was later disproven [22]. Further studies showed that vanoxerine dissipates the membrane electric potential, and has downstream impacts on efflux and membrane transport [22]. While the current work focused on the testing of vanoxerine and its analogues for use against *M. tuberculosis*, the repurposing of vanoxerine has also been explored for use against Streptococcus [23], highlighting the wider applicability of this research.

A set of vanoxerine analogues was synthesised with the aim of exploring the activity landscape around this compound. In total, ten analogues (**3**–**11** and GBR12935) were tested against *M. smegmatis*, *M. bovis* BCG and *Enterococcus faecium* E745. Following the assessment of activity and identification of the most active analogue (**3**), its cellular effects were found to be similar to those exhibited by vanoxerine. Finally, the most active analogues were tested for inhibitory activity against the human dopamine transporter and the most potent analogue, compound **3**, was shown to have reduced activity. This analogue represents a starting point for a medicinal chemistry optimisation study to identify a more potent and selective antibacterial compound for future development.

## Materials and methods

### Synthesis of vanoxerine analogues

**Fig. 1:**
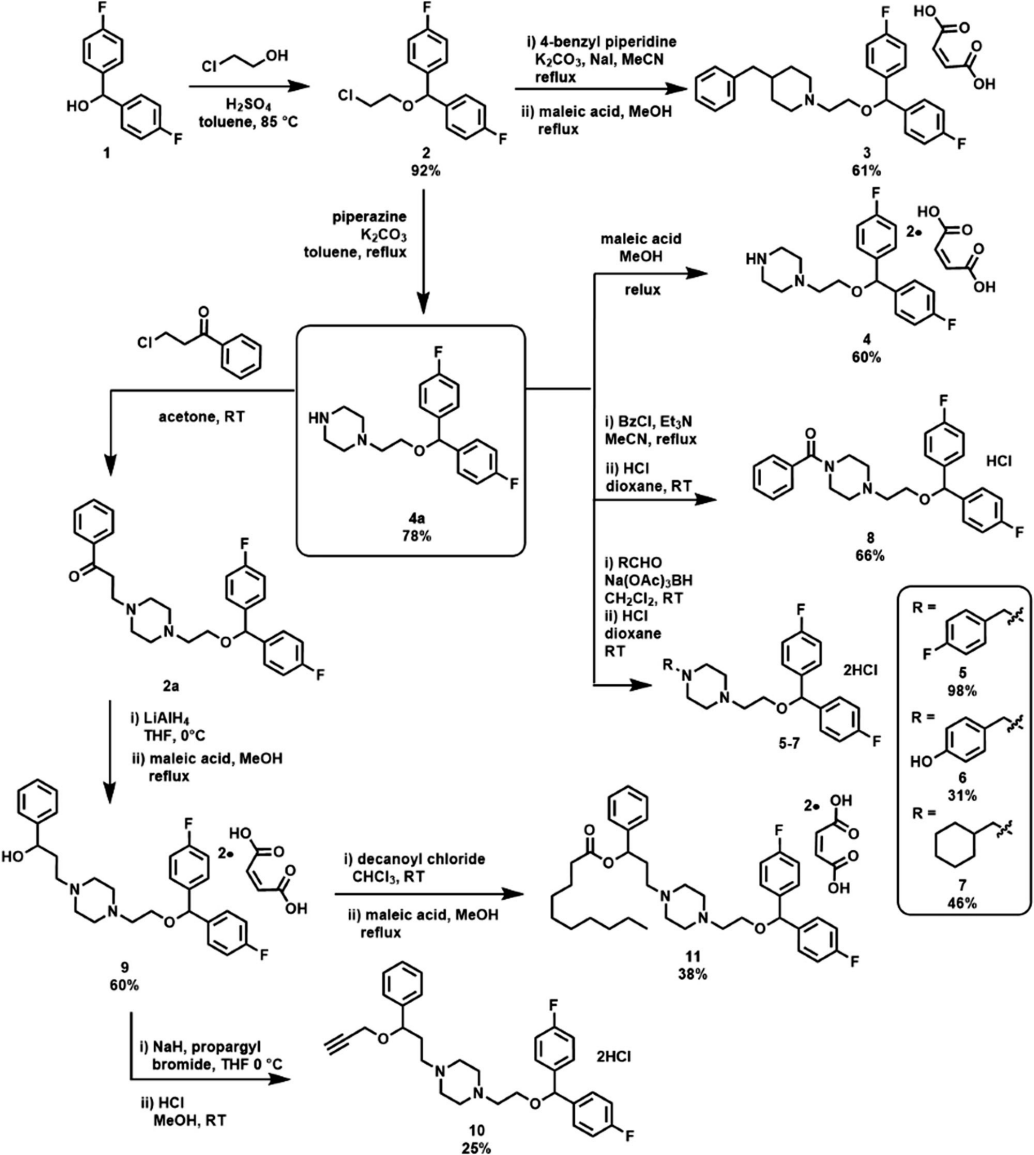
Synthesis of vanoxerine analogues **3**–**11**

Vanoxerine and GBR12935 were purchased from Adooq Bioscience and Carbosynth and were used without further purification. All other reagents were purchased from either Sigma-Aldrich (Merck), Alfa Aesar, Acros Organics, Fisher Scientific, VWR or Fluorochem, and were used as sold within the synthetic routes summarised in Fig. 1.

Vanoxerine analogues **3**–**11** were synthesised from commercially available alcohol **1** according to the Fig. 1, using methods adapted from the literature [16]. The full synthetic protocols and characterisation are provided in the supplementary material. For compounds **5**–**8**, the HCl salts were stable. Compounds **3**–**4** and **9**–**11** proved unstable to HCl; however, the maleic and bis-maleic acid salts were found to be suitable and stable salt forms for use in biological testing.

### Bacterial strains and growth conditions

The bacterial strains used were *M. smegmatis* mc^2^155, *M. bovis* BCG Pasteur and *E. faecium* E745. These were grown in either BHI broth (*E. faecium*), 7H9 broth + Tween-80 0.05% (*M. smegmatis*) or 7H9 broth + Tween-80 0.05% + oleic acid, dextrose, albumin, and catalase (OADC, *M. bovis* BCG). All strains were grown by inoculating a single colony into broth and growing at 37 °C, either shaking at 180 rpm (*E. faecium, M. smegmatis*) or statically (*M. bovis* BCG).

### MIC determination

Cultures of bacterial strains were grown to mid-log (OD_600_ = 0.5–1.0), before being diluted to a final OD_600_ of 0.05. An intermediate plate was prepared, performing a two-fold serial dilution of vanoxerine or its analogues into DMSO. Each concentration (1 µl) was transferred into a 96-well plate, followed by addition of the diluted cells (99 µl). The plate was then incubated (37 °C) for either 21 h (*M. smegmatis*), 24 h (*E. faecium*) or 144 h (*M. bovis* BCG). For *E. faecium*, the OD_600_ was measured immediately. For the mycobacteria strains, resazurin (0.02%, 30 µl per well) was added and the plate was incubated for a further 3 or 24 h (*M. smegmatis* or *M. bovis* BCG). The resazurin was converted to resorufin in metabolically active cells, hence the resorufin fluorescence (excitation at 544 nm, emission at 590 nm) was measured. Both sets of data were normalised relative to DMSO and antibiotic controls, and the percentage survival calculated.

### Ethidium bromide accumulation and efflux assay

This method has been described previously [22], but is briefly outlined here: to a 96-well assay plate, DMSO, test compounds and verapamil were added, to achieve final concentrations of 1%, 50–100 µM and 102 µM, respectively. Bacterial cultures were grown to mid-log (OD_600_ = 0.5–1.0), and then washed and resuspended in PBS + Tween-80 0.05% + 0.625 µg ml^−1^ ethidium bromide (OD_600_ = 0.8). For accumulation, glucose 0.4% was added, and the cells immediately added to the assay plate. For efflux, 50 µg ml^−1^ verapamil was added, and the culture incubated (1 h, 37 °C). The cells were then centrifuged (3000 *g*, 8 min, 4 °C) and resuspended in PBS + Tween-80 0.05% (OD_600_ = 0.8). The ethidium bromide-loaded cells were either immediately added to the assay plate, or supplemented with glucose 0.4% prior to their addition. For both assays, the fluorescence (emission at 544 nm, emission at 590 nm) was measured every 60 s for 1 h at 37 °C in a BMG Labtech POLARstar omega plate reader.

### Membrane electric potential assay

This method has been described previously [22], but is briefly outlined here: mid-log bacterial cultures were washed and resuspended in PBS + Tween-80 0.05% + 30 µM 3,3’-diethyloxacarbocyanine iodine (DiOC_2_(3)). The culture was incubated (37 °C, 2 h), before addition (99 µl) into a 96-well assay plate. Baseline fluorescence readings (excitation at 485 nm, emission at 520 and 620 nm) were taken every 90 s for 9 min, in a BMG Labtech POLARstar omega plate reader at 37 °C. DMSO, CCCP and test compounds were added, to achieve final concentrations of 1%, 25 µM and 100 µM, respectively. The fluorescence was read for a further 80 min.

### Dopamine transport inhibition assay

This assay was performed by Eurofins Discovery and is based on a reported method [24]. Transfected Chinese hamster ovary (CHO) cells (2.5 × 10^3^ cells/well) were incubated (90 minutes) at room temperature with [^3^H] dopamine (0.3 µM) in the absence or presence of test compounds in assay buffer (5 mM Tris-HCl pH 7.4, 7.5 mM HEPES/Tris, 120 mM NaCl, 5.4 mM KCl, 1.2 mM CaCl_2_, 1.2 mM MgSO_4_, 5.0 mM glucose and 1.0 mM ascorbic acid). Following incubation, the amount of [^3^H] dopamine was quantified using a scintillation counter (Topcount, Packard). The results were expressed as a percentage inhibition relative to the control uptake of [^3^H] dopamine. If the tested concentrations resulted in inhibition values of less than 50%, then an inhibition curve was estimated to calculate an IC_50_.

## Results

### Vanoxerine analogues displayed a range of efficacies against *M. smegmatis*, *M. bovis* BCG and *E. faecium*

Vanoxerine has shown activity against *M. tuberculosis* during in vitro testing [20, 21], but as it was developed originally as a dopamine transport inhibitor, the effects of structural modifications on its antibacterial activity are unknown. A range of vanoxerine analogues were therefore synthesised with the aim of identifying more potent analogues (Fig. 1) for future drug development efforts. The synthesised vanoxerine analogues were tested against *M. smegmatis*, *M. bovis* BCG and *E. faecium*, as these species were also previously inhibited by vanoxerine [22]. This allowed monitoring of each analogue and an assessment of whether it had retained, lost or gained activity compared to the parent compound. However, the *E. faecium* strain tested herein was a vancomycin-resistant strain, to study efficacy against this WHO high priority pathogen [25]. The MIC_99_ value of each compound was derived from the lowest concentration that reduced growth to 1% or less, relative to untreated and antibiotic-treated controls (Table 1, Supplementary Fig. S1). While MIC_99_ values are not typically used to evaluate drug efficacy against *Enterococcus* species, it was used here to allow for a direct comparison to the mycobacterial efficacy. Approximately half of the synthesised analogues retained inhibitory activity against the test mycobacterial strains; however, the majority of analogues lost activity against *E. faecium* (Table 1). Compound **3** displayed a greater inhibitory impact on both *M. bovis* BCG and *E. faecium*, compared to vanoxerine (MIC_99_ values of 50 and 44 µM, respectively), with an MIC_99_ value of 25 µM. Compounds **9,** **10**, and vanoxerine analogue GBR12935 also retained inhibitory activity, but only against *M. smegmatis* and *M. bovis* BCG, hence displaying some species selectivity (Table 1). Given several analogues retained antibacterial activity, the next step was to investigate if the cellular effects were the same as those for vanoxerine.

**Table 1:** Minimum inhibitory concentration values (MICs) of vanoxerine analogues against mycobacteria and *E. faecium*

Compound	Structure	MIC_99_ (µM)		
		*M. smegmatis*	*M. bovis* BCG	*E. faecium* E745
vanoxerine		50	50	44
GBR12935		100	100	>100
**3**		50	25	25
**4**		>100	>100	>100
**5**		>100	50	>100
**6**		>100	>100	>100
**7**		100	>100	100
**8**		>100	>100	>100
**9**		100	50	>100
**10**		100	100	>100
**11**		>100	>100	>100

*N* = 3. MIC_99_ values derived from the percentage survival at each tested concentration compared to DMSO only and rifampicin or Biocleanse controls

### Vanoxerine analogues retained their ability to inhibit mycobacterial efflux

The accumulation and efflux of ethidium bromide within mycobacteria has been widely used to study efflux and is a well characterised assay [26]. Hence, all vanoxerine analogues were tested at 100 µM against *M. smegmatis*, to determine their impact on efflux. For all compounds, the rates of ethidium bromide accumulation and efflux were calculated across a ten-minute assay window, alongside the overall percentage change in fluorescence at the end of the sixty-minute assay (Supplementary Table S1). These results indicated that the majority of analogues retained their ability to inhibit ethidium bromide efflux, apart from compound **11**, which had no effect on ethidium bromide efflux. This set of experiments was repeated in *M. bovis* BCG and gave similar results, but with lower levels of ethidium bromide accumulation and a smaller difference between analogues (Supplementary Table S2). These results loosely correlate with the MIC_99_ values found for each analogue, at least for their impact on the mycobacterial strains tested.

**Fig. 2:**
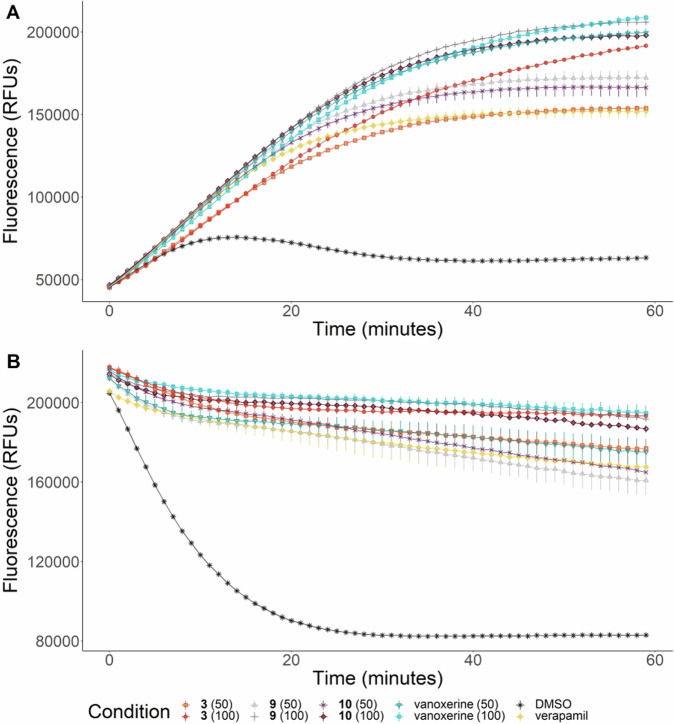
Comparison of the effect of compounds 3, 9, 10, and vanoxerine on the accumulation and efflux of ethidium bromide by *M. smegmatis*. **A** Accumulation. **B** Efflux. Compounds were tested at either 50 or 100 µM. For efflux, cells were allowed to accumulate ethidium bromide (0.625 µg ml^−1^) for 2 h. For accumulation, cells were combined with ethidium bromide (0.625 µg ml^−1^) immediately before the assay start point. In both cases, the cells were resuspended in PBS + glucose (0.4%) and added to a 96-well plate containing compounds. The fluorescence was measured every minute for 60 min. *N* = 3. DMSO = 1%, verapamil = 102 µM

The most active analogues were compounds **3,** **9** and **10**, hence, they were tested at both 50 and 100 µM in both assays, Fig. 2; this allowed a direct comparison to both vanoxerine and known efflux inhibitor verapamil. All analogues caused greater accumulation than verapamil, but only compound **9** showed similar levels of accumulation to vanoxerine, at 100 µM (Fig. 2A). In contrast, both compounds **3** and **9** caused a similar level of ethidium bromide retention to vanoxerine, suggesting they inhibit efflux as effectively (Fig. 2B). These data suggest that the vanoxerine analogues may retain a similar mechanism of action as they caused similar inhibition of ethidium bromide efflux.

### Vanoxerine analogues dissipated the *M. bovis* BCG membrane electric potential

As the analogues inhibited efflux in both *M. smegmatis* and *M. bovis* BCG, it was tested whether this behaviour was due to dissipation of the membrane electric potential (the mechanism of action of vanoxerine). Vanoxerine analogues **3**–**11** were tested against *M. bovis* BCG, using the DiOC_2_(3) voltage-sensitive dye. This dye partitions across the bacterial membrane proportional to the voltage difference, fluorescing red inside cells. Carbonyl cyanide *m*-chlorophenyl hydrazone (CCCP), a known protonophore, was used as a control to dissipate the electric potential.

**Fig. 3:**
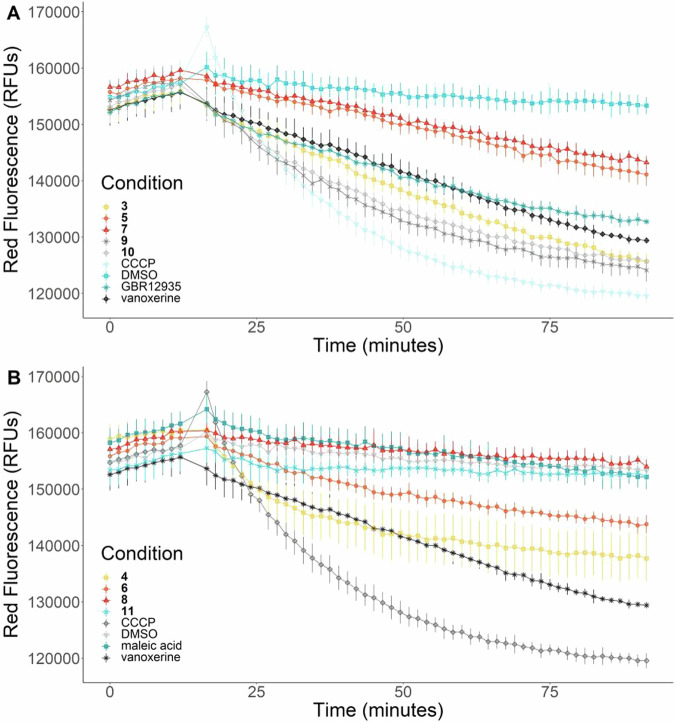
Vanoxerine analogues disrupt the *M. bovis* BCG membrane electric potential. The DiOC_2_(3) dye equilibration for 2 h before compound addition of **A** MIC-active compounds. **B** MIC-inactive compounds. All compounds were tested at 100 µM. DiOC_2_(3) dye (30 µM) was added to cells and the mixture was incubated for 2 h prior to addition of the test compounds. The test compounds were added 10 min into the read cycle. The fluorescence was measured every 90 s for 90 min. The controls (CCCP, DMSO and vanoxerine are the same across **A** and **B**). CCCP = 25 μM, DMSO = 1%. *N* = 3

In this assay, the dye was allowed to pre-equilibrate for two hours, with the fluorescence measured for ten minutes prior to compound addition. Each analogue was then added into the assay and the fluorescence response monitored over time. The results reveal that the vanoxerine analogues dissipate the membrane electric potential of *M. bovis* BCG (Fig. 3). CCCP caused the greatest loss of electric potential within the assay timeframe; however, it is known that this agent targets both the electric potential and proton gradient simultaneously, which may lead to additive effects [27]. Compounds **3,** **9** and **10** appear to have a greater impact than vanoxerine, with only compound **11** having no impact on the electric potential (Fig. 3). As the most active analogues also caused the greatest dissipation of the membrane electric potential, these results provide additional evidence for the previously proposed mode of action [22].

### Compound 3 displayed lower inhibitory activity against the dopamine transporter

If vanoxerine were to be developed as an antibacterial drug, its effects on known human targets would need to be limited to reduce drug side-effects. In relation to this, we tested if the most active synthesised analogues (compounds **3,** **9** and **10**) showed lower activity than vanoxerine against the human dopamine transporter protein (Table 2).

**Table 2:** Inhibitory impact of vanoxerine analogues on the dopamine transporter uptake

Compound	IC_50_ against dopamine transporter (nM)	Inhibition at 100 nM (%)
**V**anoxerine	1.9–3.0	N/A
**3**	140	35.8
**9**	<100	98.2
**10**	<100	95.0

*N* = 3

In this assay, five concentrations of each analogue were tested, in triplicate, ranging from 10 µM to 100 nM (Supplementary Fig. S2). The highest tested concentration of 10 µM was based on the vanoxerine concentration used to determine basal control activity when dopamine uptake is blocked in this assay, and matches the highest concentrations tested by Paudel et al. for other vanoxerine analogues [19]. If the percentage inhibition was lower than 50%, an IC_50_ value was calculated for the analogue. An IC_50_ value could only be calculated for compound **3**, being predicted to be 140 nM. For comparison, a wider range of vanoxerine concentrations were tested to calculate its IC_50_ value of between 1.9 and 3.0 nM (Table 2). Hence, compound **3** was between 46 and 74 times less active than vanoxerine against this known target of the drug. IC_50_ values could not be calculated for compounds **9** and **10** as they retained close to total dopamine transport inhibition at 100 nM; their IC_50_ values are thus <100 nM.

## Discussion

This study set out to generate a series of analogues of vanoxerine and test their biological effects. Ten analogues of vanoxerine were synthesised (compounds **3**–**11**) or purchased (GBR12935**)**, allowing an exploration of the biological impacts of these chemical modifications. This study identified compound **3**, being twice as active as vanoxerine against *M. bovis* BCG and *E. faecium*, and compound **9**, which had the same activity as vanoxerine against *M. bovis* BCG. All compounds that showed antibacterial activity had similar cellular effects as the parent molecule, namely dissipation of the membrane electric potential. Finally, the top three analogues were tested against the human dopamine transporter, with compound **3** being ~57 × less active compared to vanoxerine.

The most active analogue, compound **3**, has a calculated LogP (cLogP) > 6 suggesting it is highly hydrophobic, and calculated p*K*_a_ of 9.0 for the tertiary amine. For comparison, vanoxerine has a cLogP of 5.3 and p*K*_a_ of 8.2 for the tertiary amine. Hence, a greater proportion of compound **3** would be cationic at physiological pH and may more easily penetrate the hydrophobic cell envelope [28], both likely contributing to its increased cellular activity. This result fits within the general anti-tuberculosis drug landscape, where many TB drugs are more lipophilic than drugs for other clinical indications due to the requirement to penetrate the hydrophobic mycobacterial cell envelope [29, 30].

The ability of the vanoxerine analogues to inhibit ethidium bromide efflux and disrupt the membrane electric potential were confirmed in this study. These results are consistent with the recently proposed mechanism of action [22], and provide further evidence that the inhibition of growth could be linked to the dissipation of the membrane electric potential. In addition, work by Ortiz-Miravalles et al. showed vanoxerine inhibited *Streptococcus pneumoniae* and allowed SYTOX green dye to cross the cell membrane [23]. This dye can only cross the cell membrane when it is depolarised, providing further evidence that vanoxerine causes this electric potential depolarisation [23].

Compound **3** has previously been synthesised by Paudel et al. who reported an IC_50_ of 6.8 µM against the dopamine transporter [19]. This value is substantially higher than the 140 nM determined in this study; however, Paudel et al. also reported an IC_50_ value of 43 nM for vanoxerine [19], a 14–22-fold increase from our result. The differences in IC_50_ values may be due to the different cell lines that were used, Chinese hamster ovary cells herein, compared to human embryonic kidney 293 cells in Paudel et al. Alternatively, the assay conditions used may have contributed towards the differences, monitoring the uptake of 0.3 µM [^3^H] dopamine over 90 min at 22 °C herein, compared to 20 nM [^3^H] dopamine over 5 min at 37 °C [19]. Based on our data, there is a 179 × difference between the MIC_99_ value against *M. bovis* BCG and IC_50_ value against the dopamine transporter. Based on the data from Paudel et al. this difference is reduced to 3.7 × . Both differences are significant reductions on the 16,666 × −26,315 × difference in specificity shown by vanoxerine in our study. One limitation of this assay was the cytotoxicity of the analogues against CHO cells is not known at the tested concentrations; however, this would lead to an underestimation of the IC_50_, if both dopamine uptake inhibition and cell death were occurring concurrently.

Given the diphenylmethoxy group is likely the reason for its central nervous system bioactivity [31], analogues containing modifications to this group or removing it altogether could be tested to determine if they retain antibacterial activity. However, given the lower activity of GBR12935, which only removed the fluoro groups from the diphenylmethoxy moiety, this may not be a feasible route forward. The previously synthesised analogues of vanoxerine may represent an unexplored set of antibacterial compounds and display a range of physicochemical properties [16–19]. The addition of decanoyl esters to any vanoxerine analogue is unlikely to improve antibacterial activity, based on the complete loss of activity of compound **11**, despite its favourable pharmacokinetics properties [16].

This work has identified a vanoxerine analogue, compound **3**, with almost comparable antibacterial activity, alongside reduced activity against a known human target. Hence, this compound represents a viable starting point for additional medicinal chemistry efforts and continued drug development to target *M. tuberculosis*. Modifications that increase potency and reduce off-target effects should be optimised concurrently, to increase the likelihood of success.

## Supplementary information

Supplementary Material
