# Supplementary material for: Exploration of vanoxerine analogues as antibacterial agents
Source: J Antibiot (Tokyo). 2024 Oct 15;78(1):54–63. doi: 10.1038/s41429-024-00781-9 (PMC11700842; doi:10.1038/s41429-024-00781-9)
Supplement: Supplementary file 1 — Supplementary Material [file 41429_2024_781_MOESM1_ESM.pdf]

Supplementary Material

Supplementary Table S1: Rates of accumulation and efflux of ethidium bromide in *M. smegmatis* and the impact of vanoxerine analogues. All analogues were tested at 100 µM. N=3, (N=9 for DMSO, vanoxerine and verapamil). All values were rounded based on the standard deviation. Rate of accumulation from 5–14 minutes, rate of efflux from 0–9 minutes.

Compound	Rate of accumulation (RFUs/min)	% Change in fluorescence (accumulation)	Rate of efflux (RFUs/min)	% Change in fluorescence (efflux)
DMSO only	N/A	N/A	–5900 ± 800	–59 ±0.3
DMSO + glucose	800 ± 220	33 ± 11	–6500 ± 500	–61 ± 0.2
vanoxerine	3000 ± 130	299 ± 3	–1100 ± 500	–11 ± 0.5
GBR12935	1100 ± 40	90 ± 3	–2000 ± 100	–37 ± 0.2
3	3000 ± 100	286 ± 1	–1400 ± 200	–11 ± 0.1
4	2700 ± 310	57 ± 6	–4500 ± 400	–57 ± 0.1
5	1300 ± 20	175 ± 4	–1700 ± 200	–25 ± 0.2
6	1100 ± 40	75 ± 2	–2500 ± 200	–49 ± 0.3
7	1000 ± 16	94 ± 2	–2200 ± 60	–43 ± 0.2
8	600 ± 30	37 ± 2	–4300 ± 400	–63 ± 0.1
9	4500 ± 40	349 ± 1	–1100 ± 110	–11 ± 0.1
10	4500 ± 260	323 ± 7	–1200 ± 200	–13 ± 0.1
11	1000 ± 160	39 ± 2	–5600 ± 160	–59 ± 0.1
verapamil	N/A	N/A	–1400 ± 400	–21 ± 0.4
verapamil + glucose	2900 ± 140	210 ± 7	–1500 ± 400	–25 ± 0.5

Supplementary Table S2: Rates of accumulation and efflux of ethidium bromide in *M. bovis* BCG and the impact of vanoxerine analogues. All analogues were tested at 100 µM. N=3, (N=9 for DMSO, vanoxerine and verapamil). All values were rounded based on the standard deviation. Rate of accumulation from 5–14 minutes, rate of efflux from 0–9 minutes.

Compound	Rate of accumulation (RFUs/min)	% Change in fluorescence (accumulation)	Rate of efflux (RFUs/min)	% Change in fluorescence (efflux)
DMSO only	N/A	N/A	–2300 ± 500	–28 ± 0.5
DMSO + glucose	370 ± 1100	29 ± 3	–2300 ± 900	–28 ± 1
vanoxerine	490 ± 800	48 ± 3	–1100 ± 950	–15 ± 1
GBR12935	160 ± 240	34 ± 2	–1300 ± 150	–16 ± 0.1
3	480 ± 300	67 ± 1	–1300 ± 200	–18 ± 6
4	800 ± 2000	46 ± 2	–1000 ± 800	–16 ± 0.4
5	130 ± 130	39 ± 1	–1500 ± 50	–20 ± 0.02
6	140 ± 480	36 ± 3	–1400 ± 330	–21 ± 0.2
7	120 ± 90	32 ± 1	–1600 ± 100	–21 ± 0.1
8	120 ± 460	26 ± 2	–1799 ± 400	–26 ± 0.2
9	1100 ± 1000	68 ± 1	–1200 ± 600	–16 ± 0.4
10	1200 ± 700	66 ± 1	–940 ± 700	–13 ± 0.3
11	400 ± 300	45 ± 1	–1900 ± 400	–28 ± 25
verapamil	N/A	N/A	–1600 ± 500	–19 ± 0.6
verapamil + glucose	640 ± 1400	52 ± 6	–1500 ± 500	–19 ± 0.3

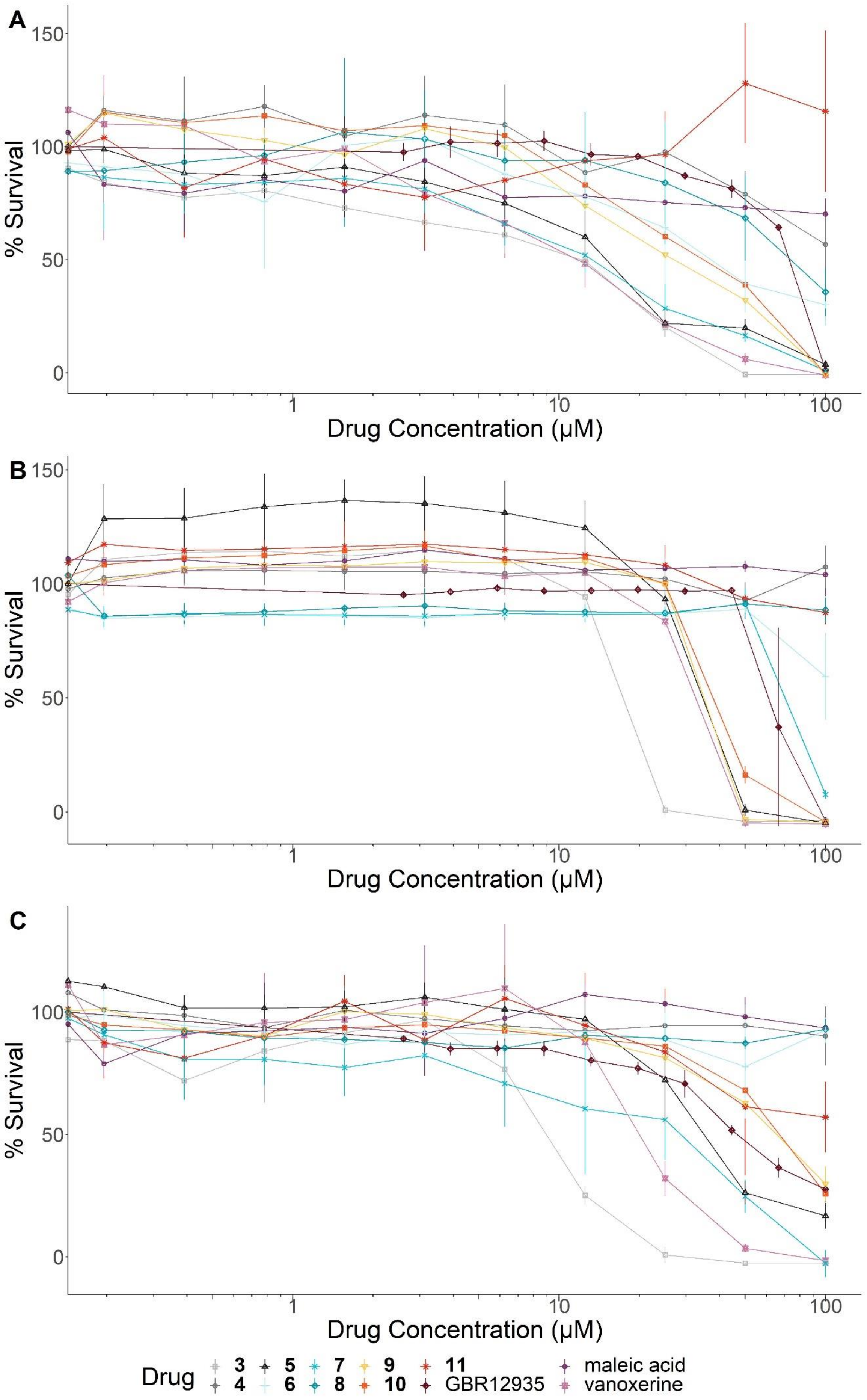

A
B
C
% Survival
150
100
50
0
1
10
100
Drug Concentration (μM)
Drug
3
4
5
6
7
8
9
10
11
GBR12935
maleic acid
vanoxerine

Supplementary Figure S1: Percentage survival curves of *M. smegmatis* (A), *M. bovis* BCG (B) and *E. faecium* E745 (C) treated with different concentrations of vanoxerine analogues. Serial dilutions (1:2) were prepared of each vanoxerine analogue, with a highest final concentration of 100 µM (and final DMSO concentration of 1%). Mid-log cultures were diluted to OD_{600} prior to addition to each plate and plates were incubated for 21 (*M. smegmatis*), 24 (*E. faecium*) or 144 (*M. bovis BCG*) hours. For *E. faecium*, the OD_{600} was measured immediately. For mycobacteria, resazurin (0.02%) was added to every well and the plate re-incubated for 3 (*M. smegmatis*) or 24 (*M. bovis* BCG) hours. The fluorescence (544 nm to 590 nm) was measured. The % survival was calculated relative to DMSO and rifampicin/Biocleanse controls. N=3.

Compound I.D.	Client Compound I.D.	IC_{50}(M)	nH	Test Concen-tration	% Inhibition of Control Values 1st	2nd	3rd	Mean	Flags 1st	2nd	3rd
**Dopamine transporter uptake (h)**											
100065870-1	HVA_482	< 1.0E-07	n/a	1.0E-07 M	86.6	100.3	98.1	95.0			
				3.0E-07 M	99.9	99.9	98.3	99.3			
				1.0E-06 M	97.1	99.4	98.4	98.3			
				3.0E-06 M	98.4	96.7	76.7	90.6			
				1.0E-05 M	98.8	93.4	98.8	97.0			

A

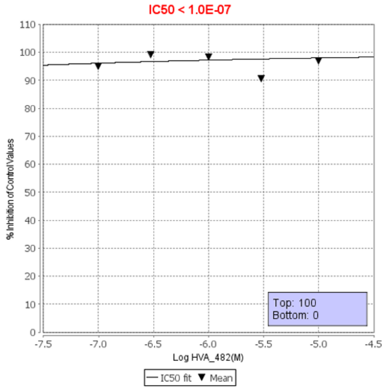

Figure 2. HVA_482 on Dopamine transporter uptake (h)

Compound I.D.	Client Compound I.D.	IC_{50}(M)	nH	Test Concen-tration	1st	2nd	3rd	Mean	Flags 1st	2nd	3rd
100065870-2	HVA_486	1.4E-07 M	n/a	1.0E-07 M	-104.6	35.8	35.9	35.8	{}		
				3.0E-07 M	77.4	77.8	74.8	76.7			
				1.0E-06 M	98.0	95.4	96.7	96.7			
				3.0E-06 M	98.4	98.4	98.1	98.3			
				1.0E-05 M	99.1	101.2	99.6	100.0			

B

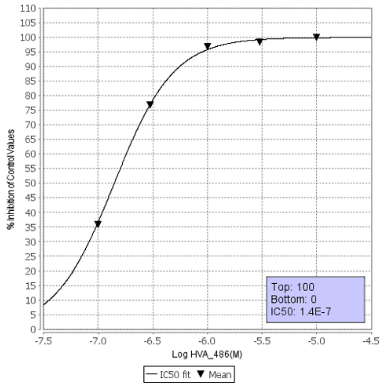

Figure 3. HVA_486 on Dopamine transporter uptake (h)

Compound I.D.	Client Compound I.D.	IC_{50}(M)	nH	Test Concen-tration	1st	2nd	3rd	Mean	Flags 1st	2nd	3rd
100065870-3	HVA_488	< 1.0E-07	n/a	1.0E-07 M	100.9	93.7	100.1	98.2			
				3.0E-07 M	99.9	98.6	99.4	99.3			
				1.0E-06 M	97.1	98.8	84.0	93.3			
				3.0E-06 M	98.1	96.8	99.6	98.2			
				1.0E-05 M	99.1	99.6	100.6	99.8			

C

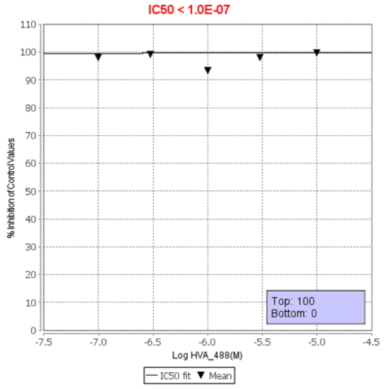

Figure 4. HVA_488 on Dopamine transporter uptake (h)

Supplementary Figure S2: Dopamine transporter uptake assays monitoring inhibition by compounds 3, 9 and 10. The uptake of [^{3}H] dopamine into Chinese hamster ovary cells, transfected with the human dopamine transporter, was used to evaluate the inhibitory activity of compounds **3**, **9** and **10**. (A) HVA_482 = compound **10**; (B) HVA_486 = compound **3**; (C) HVA_488 = compound **9**.

Chemistry – Supplementary Data and Synthetic Methods

General Experimental

All reagents were purchased from either Sigma-Aldrich (Merck), Alfa Aesar, Acros Organics, Fisher Scientific, VWR, or Fluorochem, and were used as sold. All reactions were performed under an atmosphere of argon unless otherwise stated. All solvents were purchased from one of the above suppliers and used without further drying or purification unless stated. Any solvents that were dried were stored under argon. MeOH, THF and CH_2Cl_2 were dried over 3 Å molecular sieves for at least 24 h before use. Reaction monitoring was performed using silica gel on aluminium-backed TLC plates, supplied from Merck. The plates were visualised under UV irradiation (254 nm) and $KMnO_4$ staining. Automated flash column chromatography was performed using a Teledyne ISCO CombiFlash® NextGen 300+ utilising Interchim PuriFlash® dryload columns, filled with silica gel, pore size 60 Å, 230–400 mesh particle size, 40–63 µm particle size, purchased from Sigma-Aldrich or Fluorochem.

To generate HCl salts, HCl (4 M in dioxane, equivalents determined by the number of basic nitrogen atoms) was added to a solution of the free amine (1 eq.) in dioxane (1 mL). The resulting mixture was stirred at room temperature for 1 h, before the salt was concentrated *in vacuo*.

Infra-red spectra were recorded neat (oil) or with the aid of an ATR attachment (solid) on a Perkin Elmer Spectrum 100 FT-IR spectrometer; only selected absorbances (ν_{max}, cm^{-1}) are reported. Melting points were recorded using open, glass capillaries on a Gallenkamp melting point apparatus and are uncorrected. Mass spectrometry (MS) data are reported as m/z (%) (relative intensity except in cases where only the parent ion is observed). ^{1}H-, ^{13}C- and ^{19}F-NMR spectra were recorded on a Bruker AVIII300, Bruker AVIII400, or Bruker NEO400 spectrometer in the solvents indicated and analysed using MestreNova software version 14.2.3. The solvent resonances were used as references: ^{1}H-NMR spectra: residual $CHCl_3$ (7.26 ppm), methanol-d_4 (3.31 ppm) and DMSO-d_6 (2.50 ppm), ^{13}C-NMR spectra: $CDCl_3$ (77.16 ppm), methanol-d_4 (49.00 ppm) and DMSO-d_6 (39.52 ppm), Coupling constants (J) are reported in Hz, and are reported as observed, not averaged

between the two environments that share them. The following abbreviations are used to describe multiplicity of ^{1}H-NMR resonances: m (multiplet), s (singlet), d (doublet), t (triplet), app. (apparent). The distinction between multiplet and stack is as follows: a multiplet is a single environment that is too convoluted to establish its multiplicity correctly, a stack is where multiple environments overlap, and their fidelity is lost.

1-[Bis(4-fluorophenyl)methoxyl]-2-chloroethane (2)[1]

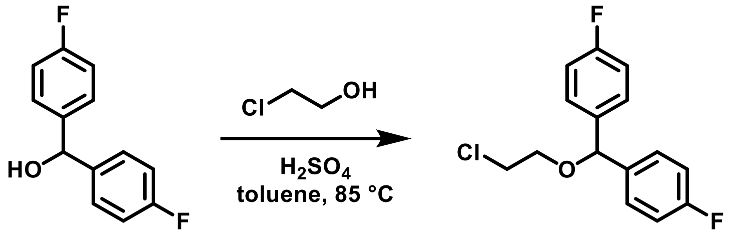

1 **2**

A solution of 4,4'-difluorobenzhydrol (2.0 g, 9.1 mmol), 2-chloroethanol (18.5 mL), *p*-toluenesulfonic acid (1.7 g, 9.1 mmol) and $MgSO_4$ (120 mg) was heated at 85˚C for 14 h. The reaction mixture was then cooled to 25˚C, diluted with hexane (12 mL), washed with saturated aqueous sodium bicarbonate solution (12 mL) and stirred for 30 min. The aqueous layer was separated and extracted with hexane (2 × 12 mL). The combined organic extracts were dried over $MgSO_4$, filtered and concentrated under reduced pressure to afforded of 1-[bis(4-fluorophenyl)methoxyl]-2-chloroethane **2** as a yellow liquid (2.41 g, 94% yield). ^{1}H NMR (300 MHz, $CDCl_3$): δ_H 7.29 – 7.16 (m, 4H), 7.05 – 6.85 (m, 4H), 5.32 (s, 1H), 3.71 – 3.52 (stack, 4H); ^{13}C NMR (400 MHz, $CDCl_3$): δ_C 162.3 (d, $J_{C–F}$ = 246 Hz), 137.3 (d, $J_{C–F}$ = 3.3 Hz), 128.6 (d, $J_{C–F}$ = 8.3 Hz), 115.4 (d, $J_{C–F}$ = 21.5 Hz), 82.7, 69.0, 43.0; LRMS (ESI+): 282, $[M + H]^+$.

4-Benzyl-1-(2-(bis(4-fluorophenyl)methoxy)ethyl)piperidine maleate (3)

---

[1]**Lewis DB, Matecka D. Zhang Y, Hsin I-W, Dersch CM, Stafford D, Glowa JR, Rothman RB, Rice KC.** Oxygenated Analogues of 1-[2-(Diphenylmethoxy)ethyl]- and 1-[2-[Bis(4-fluorophenyl)methoxy]ethyl]-4-(3-phenylpropyl)piperazines (GBR 12935 and GBR 12909) as Potential Extended-Action Cocaine-Abuse Therapeutic. *J. Med. Chem.* 1999;42:5029 – 5042. https://doi.org/10.1021/jm990291q.

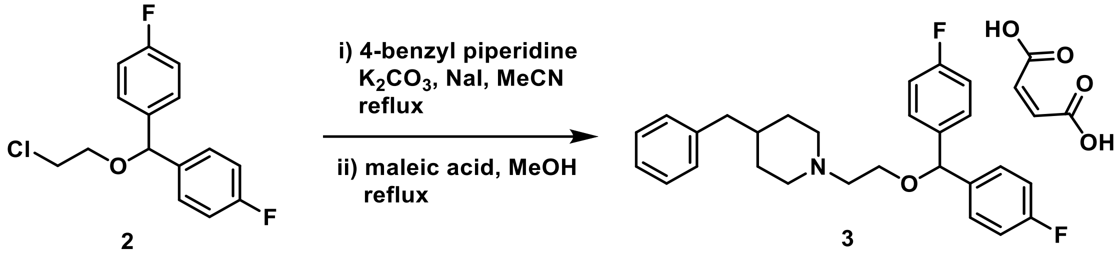

Chloride **2** (250 mg, 0.9 mmol) and 4-benzylpiperidine (233 µL, 1.3 mmol) were dissolved in MeCN (12 mL). K_2CO_3 (244 mg, 1.8 mmol) and NaI (*ca* 50 mg) were added, and the resulting suspension was stirred at reflux for 24 h. The resulting mixture was concentrated under reduced pressure and the residue purified by flash column chromatography (0–5% MeOH in CH_2Cl_2) to afford the alkylated piperidine as a clear oil which was dissolved in MeOH (10 mL) and treated with maleic acid (66 mg, 0.6 mmol, 1.1 equiv.). The resulting solution was heated at reflux for 1 h, and then cooled to room temperature and concentrated under reduced pressure to afford the maleic acid salt **3** as a clear viscous oil (214 mg, 44%). IR: ν_{max} (cm^{-1}) 3028, 2927, 2658, 1725, 1602, 1505, 1219, 823; ^{1}H NMR (400 MHz, CD_3OD) δ_H 7.43 – 7.33 (m, 4H), 7.32 – 7.23 (m, 2H), 7.22 – 7.13 (stack, 3H), 7.11 – 7.00 (m, 4H), 6.24 (s, 2H), 5.51 (s, 1H), 3.76 (app. t, *J* = 4.9 Hz, 2H), 3.55 – 3.52 (m, 2H), 3.39 – 3.37 (m, 2H), 2.98 (s, 2H), 2.61 (d, *J* = 6.8 Hz, 2H), 1.86 (d, *J* = 13.5 Hz, 3H), 1.57 – 1.47 (m, 2H), exchangeable protons not observed; ^{13}C NMR (101 MHz, DMSO-d_6) δ_C 166.9 (C=O), 161.5 (d, $J_{C–F}$ = 243.8 Hz, C), 139.3 (C), 137.8 (C), 132.5 (CH), 129.1 (CH), 128.7 (d, $J_{C–F}$ = 8.2 Hz, CH), 128.3 (CH), 126.1 (CH), 115.3 (d, $J_{C–F}$ = 21.4 Hz, CH), 81.3 (CH), 62.6 (CH_2), 55.5 (CH_2), 51.4 (CH_2), 41.4 (CH_2), 34.6 (CH), 28.7 (CH_2); ^{19}F NMR (377 MHz, DMSO-d_6) δ_F –114.91; HRMS *m/z* (TOF, ASAP+) calculated for $C_{27}H_{30}F_2NO$ [M + H]$^+$ 422.2295, found 422.2293.

1-(2-(bis(4-fluorophenyl)methoxy)ethyl)piperazine dimaleate (4)

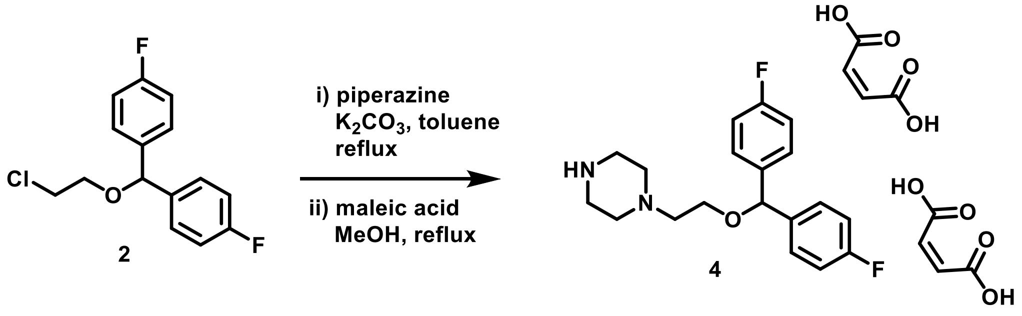

A mixture of piperazine (305 mg, 3.5 mmol) and K_2CO_3 (367 mg, 2.7 mmol) was suspended in toluene (10 mL) and heated at reflux. Chloride **2** (250 mg, 0.9 mmol) was added dropwise over 5 min and the resulting mixture was stirred. After 18 h, water (10 mL) and CH_2Cl_2 (10 mL) were added, and the layers were separated. The aqueous layer was extracted with CH_2Cl_2 (3 × 10 mL). The combined organic layers were extracted with aqueous citric acid solution (3 M, 3 × 10 mL). The combined aqueous layers were basified (~pH 8) by the addition of 25% aqueous NH_4OH solution (10 mL) and finally extracted with CH_2Cl_2 (3 × 10 mL). The combined organic layers were dried over $MgSO_4$, filtered, and concentrated under reduced pressure. The resulting oil was dissolved in MeOH (3 mL) and the solution was heated at reflux. Maleic acid (226 mg, 1.9 mmol) was added and the solution allowed to cool slowly to RT, affording a white precipitate which was filtered under vacuum, washed with petroleum ether (bp. 40 – 60°C, 2 × 20 mL) and air-dried for 48 h to afford the bis maleic acid salt **4** as a white solid (183 mg, 37%). IR: ν_{max} (cm^{-1}) 3028, 3013, 1572, 1506, 1459, 1353, 1091; ^1H NMR (400 MHz, DMSO-d_6) δ_H 7.48 – 7.32 (m, 4H), 7.23 – 7.07 (m, 4H), 6.14 (s, 4H), 5.51 (s, 1H), 3.50 (t, J = 5.5 Hz, 2H), 3.11 (app. t, J = 5.1 Hz, 4H), 2.72 (stack, 6H), 2 exchangeable protons not observed; ^{13}C NMR (101 MHz, DMSO-d_6) δ_C 167.0 (C=O), 161.4 (d, J_{C-F} = 243.4 Hz, C), 138.5 (d, J_{C-F} = 3.0 Hz, C), 133.4 (CH), 128.5 (d, J_{C-F} = 8.2 Hz, CH), 115.2 (d, J_{C-F} = 21.2 Hz, CH), 81.1 (CH), 65.8 (CH_2), 56.6 (CH_2), 49.5 (CH_2), 42.8 (CH_2); ^{19}F NMR (377 MHz, DMSO-d_6) δ_F –115.14; HRMS m/z (TOF, ASAP+) calculated for $C_{19}H_{22}F_2N_2O$ [M + H]$^+$ 333.1778, found 333.1774.

1-(2-(Bis(4-fluorophenyl)methoxy)ethyl)-4-(4-fluorobenzyl)piperazine (5)

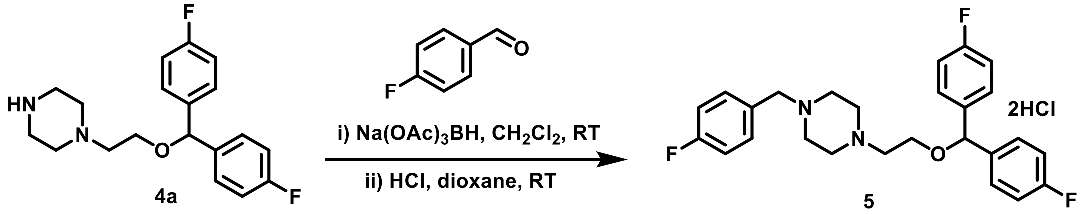

4-Fluorobenzaldehyde (16 µL, 0.2 mmol) and $Na(AcO)_3BH$ (60 mg, 0.3 mmol) were added to a solution of 1-(2-(bis(4-fluorophenyl)methoxy)ethyl)piperazine (50 mg, 0.2 mmol) in CH_2Cl_2 (0.8 mL). The reaction mixture was stirred at room temperature overnight and then partitioned between CH_2Cl_2 (5 mL) and water (5 mL). The aqueous layer was extracted with CH_2Cl_2 (5 mL). The combined organic layers were

washed with brine, dried over magnesium sulfate, filtered, and concentrated under reduced pressure to afford compound **5** as a colourless oil (65 mg, 98%). IR ν_{max} (film)/cm^{-1}: 2923, 2662, 2359, 1658, 1602, 1507, 1441, 1295, 1219, 1155, 1102, 1014, 964, 911, 824, 753, 665; ^{1}H NMR (400 MHz, $CDCl_3$): δ_H 7.39 – 7.23 (stack, 7H), 7.06 – 6.97 (stack, 5H), 5.35 (s, 1H), 3.63 – 3.61 (m, 2H), 3.50 (br s, 2H), 2.73 (br s, 2H), 2.70 – 2.41 (stack, 8H); ^{13}C NMR (101 MHz, $CDCl_3$): δ_C 162.4 (d, $J_{C–F}$ = 246 Hz, C), 161.3 (d, J_{C-F} = 244 Hz, C),150.5 (C), 137.8 (C), 130.7 (CH), 128.6 (CH), 115.4 (CH), 115.1 (d, $J_{C–F}$ = 3.9 Hz, CH), 82.6 (CH), 66.7 (CH_2), 62.1 (CH_2), 57.7 (CH_2), 53.5 (CH_2), 52.7 (CH_2); HRMS (ESI+): Calculated for $C_{26}H_{28}F_3N_2O$, 441.2160 $[M + H]^+$, found 441.2154.

The dihydrochloride salt was synthesised according to the general procedure: ^{1}H NMR (300 MHz, $CDCl_3$): δ_H 7.80 – 7.62 (m, 2H), 7.53 – 7.34 (m, 4H), 7.24 (t, J = 8.6 Hz, 2H), 7.08 (t, J = 8.6 Hz, 4H), 5.58 (s, 1H), 4.49 (s, 2H), 4.01 – 3.44 (stack, 12H), exchangeable hydrogens not observed.

4-((4-(2-(Bis(4-fluorophenyl)methoxy)ethyl)piperazin-1-yl)methyl)phenol (6)

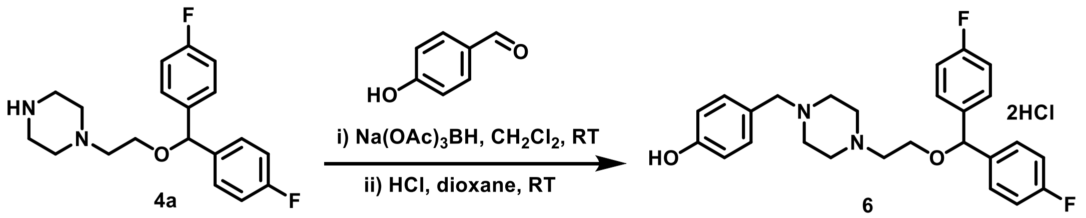

4-Hydroxybenzaldehyde (18 mg, 0.15 mmol) and $Na(AcO)_3BH$ (64 mg, 0.30 mmol) were added to a solution of 1-(2-(bis(4-fluorophenyl)methoxy)ethyl)piperazine (50 mg, 0.15 mmol) in CH_2Cl_2 (0.75 mL). The reaction mixture was stirred at room temperature for 2 hours. The resulting mixture was partitioned between CH_2Cl_2 (5 mL) and water (5 mL). The aqueous layer was extracted with CH_2Cl_2 (5 mL). The combined organic layers were washed with brine, dried over magnesium sulfate, filtered, and concentrated under reduced pressure to afford a colourless oil, which was purified by flash column chromatography (SiO_2, 95:5 CH_2Cl_2:MeOH) to afford phenol **6** as a colourless oil (20 mg, 31%). IR ν_{max} (film)/cm^{-1}: 2669, 2365, 1722, 1603, 1507, 1447, 1271, 1218, 1177, 1155, 1094, 1014, 921, 826, 750, 665; ^{1}H NMR (400 MHz, $CDCl_3$): δ_H 7.30 – 7.24 (m, 4H), 7.11 – 6.92 (stack, 6H), 6.65 – 6.56 (m, 2H), 5.32 (s, 1H), 3.57 (t, J = 5.8 Hz, 2H), 3.51 (s, 2H), 2.70 (t, J = 5.8 Hz, 2H), 2.62

(stack, 8H), OH not observed; ^{13}C NMR (101 MHz): δ_C 162.1 (d, $J_{C\text{–}F}$ = 250 Hz), 155.5, 137.8, 131.0, 128.5 (d, $J_{C\text{–}F}$ = 8.2 Hz), 115.4, 115.2, 82.6, 66.7, 62.3, 57.7, 53.3, 52.3, one quaternary aromatic carbon not observed; HRMS (ESI+): Calculated for $C_{26}H_{29}F_2N_2O_2$, $[M + H]^+$ 439.2200, found 439.2197.

The dihydrochloride salt was synthesised according to the general procedure: ^{1}H NMR (300 MHz, $CDCl_3$): δ_H 7.50 – 7.35 (stack, 6H), 7.13 – 7.01 (m, 4H), 6.94 – 6.84 (m, 2H), 5.58 (s, 1H), 4.39 (s, 2H), 3.85 – 3.72 (m, 4H), 3.75 – 3.70 (m, 8H), exchangeable hydrogens not observed.

1-(2-(Bis(4-fluorophenyl)methoxy)ethyl)-4-(cyclohexylmethyl)piperazine (7)

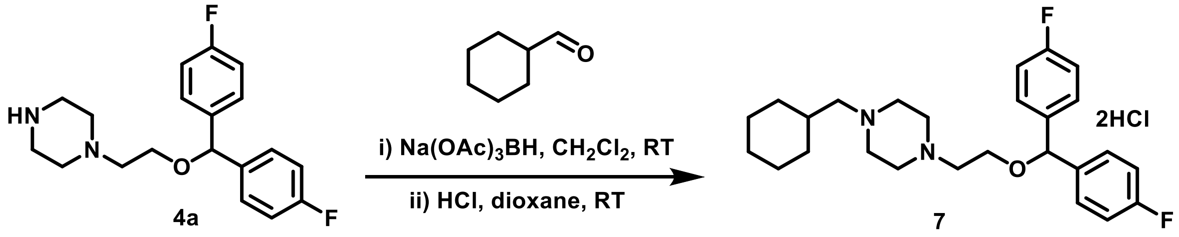

Cyclohexanecarboxaldehyde (18 μL, 0.15 mmol) and $Na(AcO)_3BH$ (60 mg, 0.30 mmol) were added to a solution of 1-(2-(bis(4-fluorophenyl)methoxy)ethyl)piperazine (50 mg, 0.15 mmol) in CH_2Cl_2 (0.75 mL). The reaction mixture was stirred at room temperature for 2 hours. The resulting mixture was partitioned between CH_2Cl_2 (5 mL) and water (5 mL). The aqueous layer was extracted with CH_2Cl_2 (5 mL). The combined organic layers were washed with brine, dried over magnesium sulfate, filtered, and concentrated under reduced pressure to afford a colourless oil, which was purified by flash column chromatography (SiO_2, 95:5 CH_2Cl_2:MeOH) to afford piperazine **7** as a yellow oil (30 mg, 46%). IR ν_{max} (film)/cm^{-1}: 2925, 2852, 1659, 1603, 1506, 1448, 1221, 1096, 1014, 976, 920, 833, 749, 664; ^{1}H NMR (400 MHz, $CDCl_3$): δ_H 7.32 – 7.23 (m, 4H), 7.07 – 6.94 (m, 4H), 5.34 (s, 1H), 3.57 (t, J = 6.1 Hz, 2H), 2.67 (t, J = 6.1 Hz, 2H), 2.47 (br s, 8H), 2.12 (d, J = 7.1 Hz, 2H), 1.87 – 1.59 (stack, 5H), 1.51 – 1.44 (m, 1H), 1.38 – 1.04 (m, 3H), 0.98 – 0.70 (m, 2H); HRMS (ESI+): Calculated for $C_{26}H_{35}F_2N_2O$, $[M + H]^+$ 429.2720, found 429.2717.

The dihydrochloride salt was synthesised according to the general procedure: ^{1}H NMR (300 MHz, $CDCl_3$): δ_H 7.41 – 7.28 (m, 4H), 7.07 – 6.88 (m, 4H), 5.50 (s, 1H), 3.94 – 3.66 (stack, 8H), 3.55 – 3.37 (stack, 4H), 3.05 (d, J = 6.6 Hz, 2H), 1.91 –

1.51 (stack, 6H), 1.43 – 1.05 (stack, 3H), 1.07 – 0.81 (m, 2H), exchangeable hydrogens not observed.

(4-(2-(Bis(4-fluorophenyl)methoxy)ethyl)piperazin-1-yl)(phenyl)methanone (8)

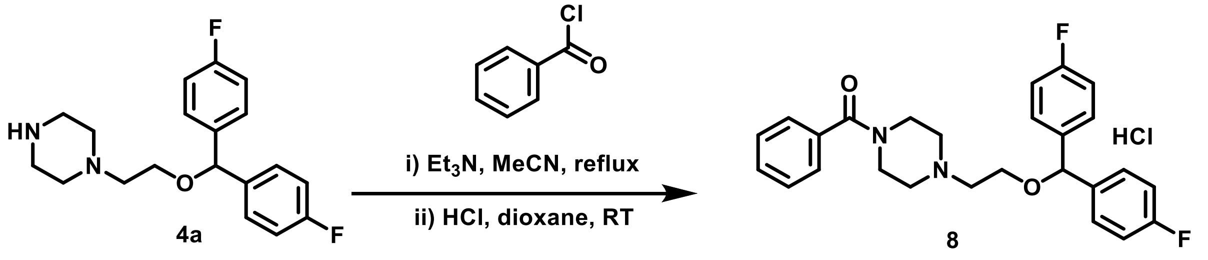

Triethylamine (25 µL, 0.2 mmol) and benzoyl chloride (21 mg, 0.2 mmol) were added sequentially to a solution of 1-(2-(bis(4-fluorophenyl)methoxy)ethyl)piperazine (50 mg, 0.2 mmol) in acetonitrile (0.3 mL). The mixture was heated at reflux for 3 h. The resulting mixture was partitioned between ethyl acetate (5 mL) and water (5 mL). The aqueous layer was extracted with ethyl acetate (3 x 5 mL). The combined organic layers were washed with brine, dried over magnesium sulfate, filtered, and concentrated under reduced pressure to afford a yellow oil, which was purified by flash column chromatography (SiO_2, 95:5 CH_2Cl_2:MeOH) to afford amide **8** as a yellow oil (40 mg, 66%). IR ν_{max} (film)/cm^{-1}: 1708, 1632, 1507, 1425, 1286, 1219, 1108, 997, 927, 833, 746, 710, 665; ^{1}H NMR (400 MHz, $CDCl_3$): δ_H 8.15 – 8.05 (m, 2H), 7.65 – 7.56 (m, 1H), 7.53 – 7.36 (m, 2H), 7.33 – 7.22 (m, 4H), 7.06 – 6.94 (m, 4H), 5.33 (s, 1H), 3.88 (s, 2H), 3.64 (t, J = 5.6 Hz, 2H), 3.53 (s, 2H), 2.81 (t, J = 5.6 Hz, 2H), 2.73 (s, 2H), 2.60 (s, 2H); ^{13}C NMR (101 MHz, $CDCl_3$): δ_C 170.4 (C=O), 162.7 (d, $J_{C\text{–}F}$ = 240 Hz, C), 137.6 (C), 135.5 (C), 130.0 (CH), 129.9 (CH), 128.5 (CH), 127.1 (CH), 115.3 (d, $J_{C\text{–}F}$ = 21.3 Hz, CH), 82.7 (CH), 66.3 (CH_2), 57.5 (CH_2), 53.5 (CH_2), 52.9 (CH_2), HRMS (ESI+): Calculated for $C_{26}H_{27}F_2N_2O_2$, $[M + H]^+$ 437.2042, found 437.2041.

The hydrochloride salt was synthesised according to the general procedure: ^{1}H NMR (300 MHz, $CDCl_3$): δ_H 8.08 – 7.98 (m, 2H), 7.48 – 7.33 (stack, 7H), 7.01 – 6.92 (stack, 3H), 5.30 (s, 1H), 3.83 (br s, 4H), 3.59 (t, J = 5.6 Hz, 2H), 3.49 (br s, 2H), 2.77 (t, J = 5.6 Hz, 2H), 2.62 (stack, 4H).

3-(4-(2-(Bis(4-fluorophenyl)methoxy)ethyl)piperazin-1-yl)-1-phenylpropan-1-ol (9)

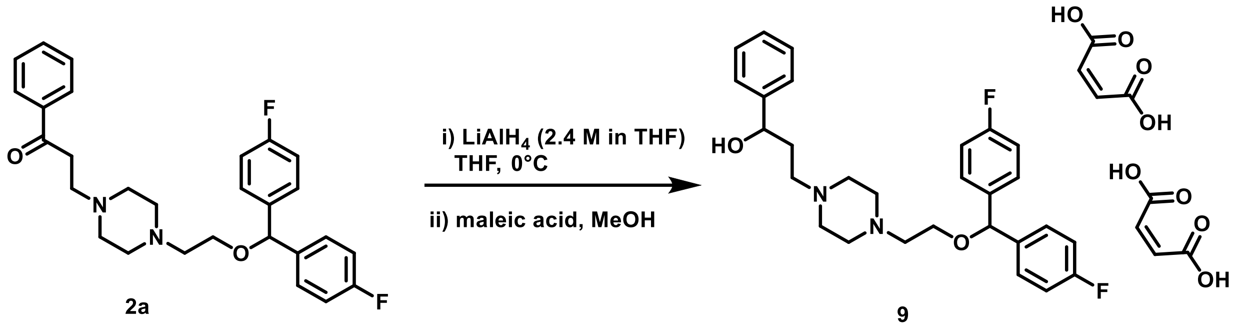

2a 9

A solution of ketone **2a**[1] (1.0 g, 2.2 mmol) in THF (10 mL) was added over 30 min to a cold (ice/water bath) solution of $LiAlH_4$ (2.4 M in THF, 720 µL, 1.7 mmol) in THF (10 mL). The resulting solution was stirred. After 30 min, aqueous NaOH solution (2 M, 5 mL) was added over 5 min and the resulting precipitate was filtered through Celite, washing with THF (50 mL). The filtrate was concentrated under reduced pressure and the residue was purified by flash column chromatography (SiO_2, 0–5% MeOH in CH_2Cl_2) to afford alcohol **9a** as a clear oil (805 mg, 80%). A solution of alcohol **9a** (95 mg, 0.2 mmol) in MeOH (2 mL) was heated at reflux. Maleic acid (61 mg, 0.5 mmol) was added and the solution allowed to cool slowly to RT, affording a white precipitate which was filtered under vacuum, washed with petroleum ether (bp. 40 – 60°C, 2 × 20 mL) and air-dried for 48 h to afford the bis maleic acid salt **9** as a white solid (86 mg, 60%). m.p.: 176 – 177°C; IR: ν_{max} (cm^{-1}) 3230, 3006, 2970, 1737, 1572, 1506, 1475, 1438, 1356, 1216; ^1H NMR (400 MHz, DMSO-d_6) δ_H 7.41 – 7.31 (stack, 8H), 7.29 – 7.22 (m, 1H), 7.21 – 7.12 (m, 4H), 6.15 (s, 4H), 5.52 (s, 1H), 4.64 – 4.61 (m, 1H), 3.52 (t, J = 5.3 Hz, 2H), 3.17 – 2.67 (stack, 11H), 1.94 – 1.86 (m, 2H), 2 exchangeable protons not observed; ^{13}C NMR (101 MHz, DMSO-d_6) δ_C 167.0 (C=O), 161.4 (d, $^1J_{C–F}$ = 243.4 Hz, C), 145.2 (C), 138.4 (d, $^4J_{C–F}$ = 3.1 Hz, C), 133.2 (CH), 128.6 (d, $^3J_{C–F}$ = 8.1 Hz, CH), 128.2 (CH), 127.2 (CH), 125.7 (CH), 115.2 (d, $^2J_{C–F}$ = 21.4 Hz, CH), 81.1 (CH), 70.1 (CH), 65.3 (CH_2), 55.9 (CH_2), 53.5 (CH_2), 50.8 (CH_2), 50.2 (CH_2), 33.8 (CH_2); ^{19}F NMR (377 MHz, DMSO-d_6) δ_F –115.09; HRMS m/z (TOF, ASAP+) calculated for $C_{28}H_{33}F_2N_2O_2$ [M + H]$^+$ 467.2510, found 467.2509.

3-(4-(2-(bis(4-fluorophenyl)methoxy)ethyl)piperazin-1-yl)-1-phenylpropan-1-ol dihydrochloride (10)

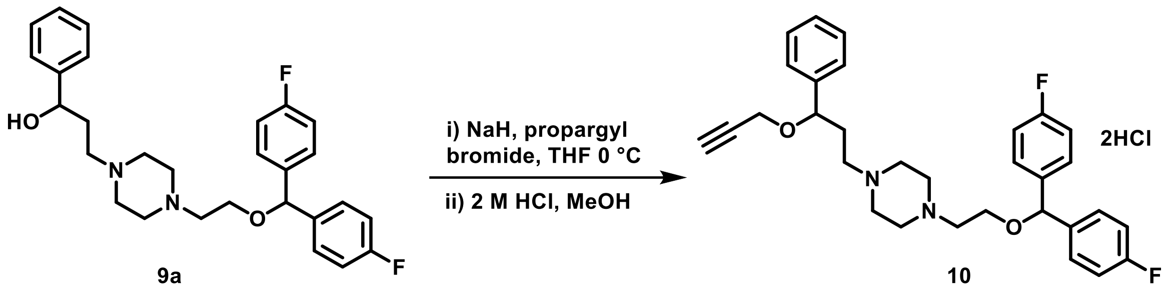

NaH (60% suspension in mineral oil, 38 mg, 1.0 mmol) and propargyl bromide (80% solution in toluene, 86 µL, 0.8 mmol) were added sequentially to a solution of alcohol **9a** (300 mg, 0.6 mmol) in THF (5 mL) at 0°C. The resulting suspension was allowed to warm to RT over 1 h. The reaction mixture was then cooled using an ice/water bath, and the reaction quenched by the addition of saturated aqueous NH_4Cl solution (2 mL), and then poured into saturated $NaHCO_3$ solution (10 mL). EtOAc (15 mL) was added, the layers were separated, and the aqueous layer was extracted with EtOAc (3 × 15 mL). The combined organic layers were washed with brine (20 mL), dried over $MgSO_4$, filtered, and purified by flash column chromatography (SiO_2, 0–5% MeOH in CH_2Cl_2). The resulting oil was dissolved in Et_2O (5 mL), treated with an excess of HCl (2 M in Et_2O, 2 mL) and stirred at RT for 10 min before concentrating and drying under high vacuum to afford the dihydrochloride salt **10** as a white solid (94 mg, 25%). IR: ν_{max} (cm^{-1}) 3346, 3008, 2970, 2946, 2570, 2323, 1737, 1601, 1503, 1215; ^{1}H NMR (400 MHz, DMSO-d_6) δ_H 12.00 (br s, 2H), 7.48 (dd, J = 8.4, 5.5 Hz, 4H), 7.41 – 7.31 (stack, 4H), 7.29 – 7.22 (m, 1H), 7.22 – 7.10 (m, 4H), 5.60 (s, 1H), 4.67 (app. dd, J = 8.7, 4.0 Hz, 1H), 3.91 – 3.12 (stack, 17H), 2.16 – 1.97 (m, 2H); ^{13}C NMR (101 MHz, DMSO-d_6) δ_C 162.7 (d, $^1J_{C–F}$ = 243.6 Hz, C), 145.1 (C), 137.9 (d, $^4J_{C–F}$ = 2.9 Hz, C), 128.7 (d, $^3J_{C–F}$ = 8.3 Hz, CH), 128.1 (CH), 127.1 (CH), 125.6 (CH), 115.3 (d, $^3J_{C–F}$ = 21.3 Hz, CH), 81.5 (stack, CH), 69.7 (CH), 62.8 (CH_2), 55.16 (CH_2), 55.17 (CH_2), 53.7 (CH_2), 48.6 (CH_2), 48.1 (CH_2), 32.8 (CH_2), alkyne C not observed; ^{19}F NMR (377 MHz, DMSO-d_6) δ_F –114.91; HRMS m/z (TOF, ASAP+) calculated for $C_{31}H_{34}F_2N_2O_2$ $[M + H]^+$ 505.2667, found 505.2671.

3-(4-(2-(bis(4-fluorophenyl)methoxy)ethyl)piperazin-1-yl)-1-phenylpropyl decanoate dimaleate (11)

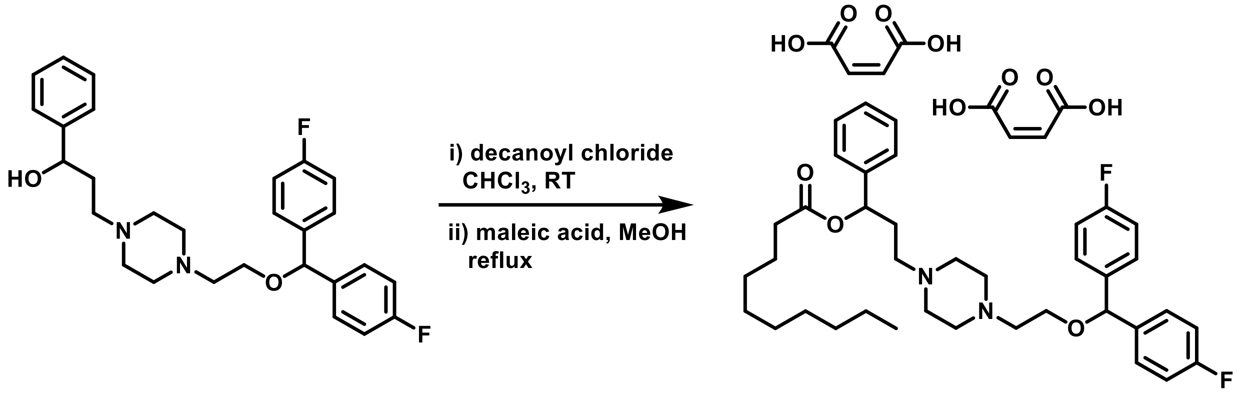

Decanoyl chloride (70 µL, 0.3 mmol) was added dropwise over 10 min to a solution of alcohol **9a** (100 mg, 0.2 mmol) in $CHCl_3$ (3 mL). The resulting solution was stirred for 16 h at RT and then concentrated under reduced pressure. The residue was dissolved in MeOH (2 mL) and the solution heated to reflux, and treated with maleic acid (64 mg, 0.5 mmol). The solution was cooled slowly and concentrated under reduced pressure. The residue was triturated with water (2 × 10 mL) and then Et_2O (3 × 10 mL) before drying under high vacuum to afford the bis maleic acid salt **11** as a viscous beige solid (70 mg, 38%, containing 8.2% excess maleic acid as determined by ^{1}H-NMR spectroscopy); IR: ν_{max} (cm^{-1}) 3031, 2923, 2854, 1734, 1604, 1564, 1506, 1218; ^{1}H NMR (400 MHz, DMSO-*d*$_6$) δ_H 7.50 – 7.42 (m, 4H), 7.42 – 7.28 (stack, 5H), 7.21 – 7.13 (m, 4H), 6.27 (s, 4H), 5.77 (dd, *J* = 8.7, 4.8 Hz, 1H), 5.59 (s, 1H), 3.73 – 3.69 (m, 2H), 3.67 (s, 2H), 3.58 – 3.36 (stack, 4H), 3.08 (br s, 2H), 2.41 – 2.16 (m, 3H), 2.08 (s, 3H), 1.53 – 1.51 (stack, 3H), 1.28 – 1.22 (stack, 12H), 0.87 – 0.83 (stack, 4H); ^{13}C NMR (101 MHz, CD_3OD) δ_C 174.6 (C=O), 170.4 (C=O), 163.7 (d, $^1J_{C-F}$ = 245.0 Hz, C), 141.3 (C), 139.0 (d, $^4J_{C-F}$ = 3.2 Hz, C), 135.7 (CH), 130.0 (d, $^3J_{C-F}$ = 8.2 Hz, CH), 129.7 (CH), 129.3 (CH), 127.5 (CH), 116.3 (d, $^2J_{C-F}$ = 21.7 Hz, CH), 84.0 (CH), 74.9 (CH), 65.3 (CH_2), 56.8 (CH_2), 54.5 (CH_2), 52.7 (CH_2), 51.7 (CH_2), 35.2 (CH_2), 33.4 (CH_2), 33.0 (CH_2) 30.5 (CH_2), 30.4 (CH_2, 2 overlapping resonances), 30.1 (CH_2), 26.1 (CH_2), 22.3 (CH_2), 14.4 (CH_3); ^{19}F NMR (377 MHz, DMSO-*d*$_6$) δ_F –114.94; HRMS *m/z* (TOF, ASAP+) calculated for $C_{38}H_{51}F_2N_2O_3$ [M + H]$^+$ 621.3868, found 621.3873.
